# Next-Generation Vaccines Against Neglected Diseases: New Promises from Genetically Modified Live-Attenuated Parasites and RNA Vaccines

**DOI:** 10.3390/microorganisms14051112

**Published:** 2026-05-14

**Authors:** Marina Ferreira Batista-Zauli, Maria Eduarda Carvalho Guimarães Brasil, Carlos Roberto de Almeida-Júnior, Bárbara Germana Soares de Abreu, Nailma Silva Aprigio dos Santos, Mayra Fernanda Ricci, Santuza Maria Ribeiro Teixeira

**Affiliations:** 1 Departamento de Bioquímica e Imunologia, Universidade Federal de Minas Gerais, Belo Horizonte 31270-901, MG, Brazil; batistamarina.gen@gmail.com (M.F.B.-Z.); me.carvalhobrasil@gmail.com (M.E.C.G.B.); carlos.almeidajr7@gmail.com (C.R.d.A.-J.); barbaraabreu7@gmail.com (B.G.S.d.A.); nailmaaprigio@gmail.com (N.S.A.d.S.); riccimayra@gmail.com (M.F.R.); 2 Centro de Tecnologia de Vacinas (CT Vacinas), Universidade Federal de Minas Gerais, Belo Horizonte 31310-260, MG, Brazil

**Keywords:** parasitic disease, live-attenuated vaccine, RNA vaccine

## Abstract

Different protozoan parasites are the causative agents of tropical diseases, including malaria, toxoplasmosis, leishmaniasis, and Chagas disease (CD), which, altogether, affect over 300 million people throughout the world. Except for two recently approved malaria vaccines, individuals affected by or at risk of contracting any of these four diseases still experience a lack of effective treatments and vaccines. Many vaccine studies, including those that have reached clinical trials, are based on inactivated parasites, adjuvanted recombinant proteins, or viral vector vaccines. Here, we review the current advances towards the development of vaccines based on genetically modified live-attenuated parasites (GMLAP) as well as RNA formulations encoding parasite antigens. Because these are diseases caused by intracellular pathogens that depend on efficient T-cell responses for parasite control, these two new vaccine platforms have generated great expectations, since they are known to induce a robust cellular immune response. Although preclinical studies aimed at developing new malaria, toxoplasmosis, and leishmaniasis vaccines have led to significant progress that may soon result in clinical trials, advances in next-generation vaccines against CD are lagging behind. Increased collaborative efforts between research groups, governments, and the pharmaceutical industry, particularly in Africa, Asia, and Latin American countries, are urgently needed to accelerate the development of vaccines for all neglected and less-studied diseases.

## 1. Neglected Tropical Diseases and the Challenging Road Towards Vaccines Against Protozoan Parasites

**Figure 1:**
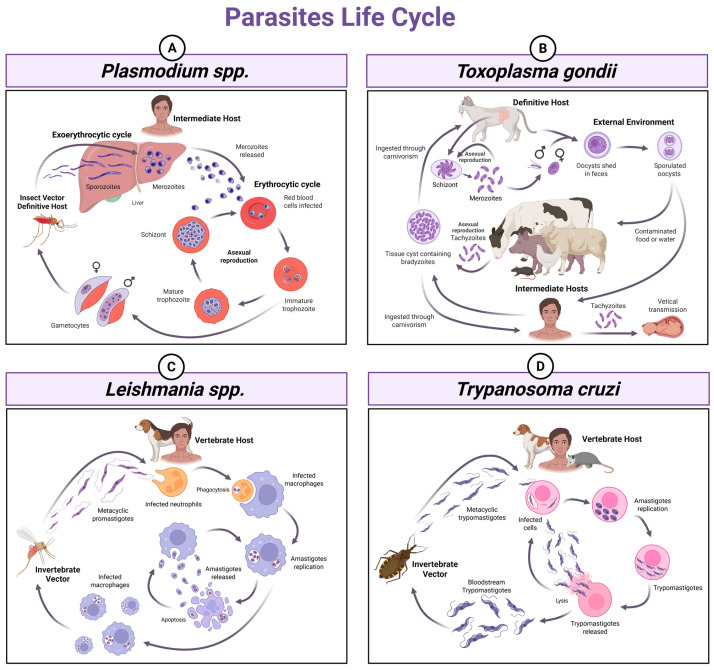
Life cycles of four major protozoan parasites that cause human disease**.** Representation of the life cycles of *Plasmodium* spp., *Toxoplasma gondii*, *Leishmania* spp., and *Trypanosoma cruzi*. *Plasmodium* spp. are transmitted by *Anopheles* mosquitoes, which inoculate sporozoites that infect hepatocytes and then initiate the erythrocytic cycle in red blood cells. A subset of blood-stage parasites differentiates into gametocytes, the sexual forms that are taken up by the mosquito during a blood meal, where fertilization and parasite development occur (**A**). *T. gondii* has felids as definitive hosts, where sexual reproduction occurs in the intestinal epithelium, resulting in the shedding of oocysts that contaminate the environment. Intermediate hosts, including humans, become infected by ingestion of oocysts or tissue cysts containing bradyzoites, whereas felids acquire infection mainly by consuming infected prey containing tissue cysts, thus completing the cycle (**B**). *Leishmania* spp. are transmitted by phlebotomine sand flies, which inject promastigotes that are internalized by macrophages and differentiate into intracellular amastigotes that replicate within host cells; these are later ingested by the sand fly, allowing parasite differentiation and continuation of the cycle in the vector (**C**). *T. cruzi* is transmitted by triatomine bugs, which deposit infective metacyclic trypomastigotes on the skin during a blood meal. Trypomastigotes invade host cells, differentiate into intracellular amastigotes, replicate, and subsequently transform into bloodstream trypomastigotes that disseminate in the mammalian host or are taken up by the insect vector during feeding (**D**). Created in BioRender. Brasil, M.E.C.G. & de Almeida-Júnior, C.R. (2026). https://BioRender.com/3afq86i.

Neglected tropical diseases (NTDs) are a group of diseases caused by various pathogens (e.g., viruses, bacteria, parasites, and fungi) that primarily affect people in impoverished communities. According to the World Health Organization (WHO), at least 1.410 billion people required healthcare support against NTDs in 2024 [1]. Among NTDs with a large impact on the population are malaria, toxoplasmosis, leishmaniasis, and CD, all of which are caused by protozoan parasites [2]. As shown in Figure 1, these diseases have a variety of hosts besides humans and are transmitted by different vectors and routes.

Malaria represents the deadliest of all NTDs, with an estimated 282 million cases reported across 80 countries that resulted in approximately 610,000 deaths in 2024 [3]. Concentrated in tropical and subtropical zones, with the heaviest burden in sub-Saharan Africa, endemic areas include regions of Oceania, Central and South America, and Southeast Asia [3]. Malaria is caused by five species of the genus *Plasmodium* (*P*): *P. falciparum*, *P. vivax*, *P. malariae*, *P. ovale*, and the zoonotic species *P. knowlesi* [4]. *P. falciparum* and *P. vivax* are the species that contribute to most of the global malaria burden in humans [3], with *P. falciparum* being the species that accounts for most severe malaria cases, whereas *P. vivax* is the species with the broadest geographic distribution outside Africa [5].

Malaria transmission occurs through the bite of an infected female *Anopheles* mosquito (Figure 1A). Malaria symptoms range from mild to life-threatening. Mild manifestations include fever, chills, and headaches. Infants, children under five years of age, pregnant women, and individuals living with HIV/AIDS are at increased risk of developing severe disease, which is characterized by hyperparasitemia and clinical complications such as severe anemia, respiratory distress, renal impairment, extreme fatigue, impaired consciousness, and convulsions, particularly in cases of cerebral malaria [3]. Antimalarial treatments include artemisinin-based combination therapies (ACTs) for *P. falciparum* infection and chloroquine for *P. vivax*, often combined with primaquine. However, these treatments are under threat due to the emergence and spread of parasite resistance [3,6]. In 2021 and 2023, respectively, two approved vaccines against *P. falciparum* malaria, RTS, S/AS01 and R21/Matrix-M, began to be distributed in African countries [7,8], but no vaccine against *P. vivax* has yet reached clinical trials.

Toxoplasmosis has a worldwide distribution, with nearly one-third of the human population infected [9,10,11]. High rates of toxoplasmosis have been observed in Africa, the Middle East, Oceania, Southeast Asia, Central Europe, Latin America, Eastern Europe, Canada, and the United States (US) [12,13]. In the US alone, it is estimated that at least 40 million people are infected [14]. The disease is caused by a single species of protozoan parasite, *Toxoplasma gondii* [15] whose population has extensive genetic diversity. Its high prevalence can be attributed to the ability of the parasite to infect all homeothermic hosts and multiple cell types [10,16]. Transmission occurs mainly through ingestion of tissue cysts in undercooked meat or sporulated oocysts in contaminated food and water, as well as in the feces from infected felines (Figure 1B) [17,18]. Most infected individuals do not develop clinical signs of the disease, but the parasite can evade the host’s inflammatory response and persist in tissues, leading to chronic infection. *T. gondii* poses a particularly high risk to pregnant women and immunocompromised individuals, such as those living with HIV, cancer patients, transplant recipients, and individuals with auto-immune diseases [14,19]. In the most severe complication, toxoplasmic encephalitis, tachyzoites may cause severe brain lesions that may be fatal if left untreated [20,21]. In pregnant women, infection is often mild, but can cause severe fetal or neonatal complications (abortion, stillbirth, hydrocephalus, intracranial calcifications, and blindness) [22]. Current antifolate therapies show limited efficacy, failing to eliminate chronic bradyzoite forms, and are often associated with significant adverse effects [20,23].

Leishmaniasis is endemic in the Americas, Africa, Europe, the Eastern Mediterranean, and Southeast Asia, where an estimated 12 million people from 99 countries are affected by some form of the disease [24]. Phlebotomine sand flies, especially species from the genera *Psychodopygus* and *Lutzomyia*, are biological vectors of *Leishmania* spp. that transmit the parasite to vertebrate hosts through the bite of infected females (Figure 1C) [25]. Leishmaniases are caused by more than 20 species of *Leishmania* and present distinct clinical forms, including visceral (VL), cutaneous (CL), mucocutaneous (MCL), and post-kala-azar dermal leishmaniasis (PKDL) [26]. Among the different clinical forms of leishmaniasis, VL is the most severe, characterized by prolonged fever, weight loss, hepatosplenomegaly, and anemia, and is associated with high mortality if untreated. It is mainly caused by *L. infantum* and *L. donovani*. CL is related to a broad range of species from the Leishmania subgenus (*L. infantum*, *L. tropica, L. major*, *L. aethiopica*, and *L. donovani*) and the Viannia subgenus (*L. braziliensis*, *L. panamensis, L. guyanensis*, and *L. peruviana*), whereas MCL is mainly caused by *L. braziliensis* and *L. panamensis*. CL forms cause skin ulcers, whereas MCL disease leads to destructive lesions of the mucosa. PKDL occurs predominantly in *L. donovani*-prevalent regions and represents a severe clinical consequence of infection [27,28]. Moreover, HIV coinfection is reported to increase disease severity and mortality, particularly in low-income endemic areas [27]. Clinical heterogeneity limits the establishment of standardized treatment protocols for leishmaniasis, and therapy is generally based on amphotericin B, antimonials, or miltefosine, with the choice depending on clinical form, parasite species, geographic region, and individual variability. However, current therapies are constrained by high toxicity, prolonged treatment regimens, and emerging drug resistance [27,28].

Chagas disease (CD) or American trypanosomiasis is endemic in most Latin American countries, where it affects an estimated 7 million people [29]. Due to increased migration, CD is now also present in the US, Canada, Europe, and Eastern Pacific countries [30,31]. CD is caused by a single species of protozoan parasite, *Trypanosoma cruzi* [32,33], whose population presents wide genetic diversity. *T. cruzi* is classified into seven discrete typing units (DTUs), designated TcI to TcVI and TcBat, which differ in their epidemiology, geographic distribution, and pathogenic potential [34,35,36]. Vector transmission occurs through direct contact with excreta (feces or urine) of infected triatomine bugs deposited during the blood meal (Figure 1D). At least 20 species from the genera *Triatoma*, *Rhodnius*, and *Panstrongylus* are relevant for *T. cruzi* transmission. Transmission also occurs through the congenital route, blood transfusion, and organ transplantation [37]. Moreover, *T. cruzi* can be transmitted by ingestion of contaminated food with bugs or their excrements, which characterize outbreaks of acute infection due to the high load of parasites ingested [38]. Although most infected individuals do not develop a serious disease, 30% of infected individuals progress from an acute phase, characterized by high parasitemia, to a chronic phase, marked by reduced circulating parasites but persistent local inflammation and progressive tissue damage [39]. Individuals in the chronic phase may be asymptomatic or develop cardiomyopathy, megasyndromes, or mixed clinical forms [35]. Only two commercially available drugs are available for CD treatment, benznidazole (BZ) and nifurtimox (NF), both of which are highly toxic and mainly effective only during the acute phase of the disease [40,41].

Except for toxoplasmosis, the four NTDs described here are caused by pathogens that present complex life cycles that alternate from vertebrate to invertebrate hosts, a situation that gained renewed importance in the climate crisis context [42]. Consistently, recent reports have shown the presence of *T. cruzi*-infected triatomine bugs near domestic urban areas in Brazil (Mato Grosso state), as well as in places in the south of the US [43,44]. Therefore, new cost-effective strategies to minimize the health impacts of parasitic diseases are essential, ranging from improved vector control to the development of novel drugs, diagnosis technologies, and vaccines [45]. Vector control strategies are often insufficient due to insecticide resistance, operational limitations, and the difficulty of eliminating vectors in complex ecological environments [46]. Efforts towards the development of new treatments for these NTDs have stagnated over the past decades, with only a few new compounds currently undergoing clinical trials [47,48].

The development of vaccines for these four NTDs faces major challenges, as it requires a comprehensive understanding of host–parasite interactions, including the components of the host immune response and the mechanisms by which parasites evade it. Except for malaria, no vaccines are currently available to prevent these NTDs. A clear understanding of the elements involved in these complex interactions, which vary greatly when the four diseases are compared and can determine the course of the disease, is only now being reached. As an example, the involvement of the innate immune system in response to these infections, including the characterization of protozoan-derived pathogen-associated molecular patterns (PAMPs) and the corresponding host pattern recognition receptors (PRRs), such as Toll-like receptors (TLRs), only came to light in the early 2000s [49].

Generally, recognition of protozoan parasite molecules by Toll-like receptors (TLRs) expressed on antigen-presenting cells is key to inducing the production of pro-inflammatory cytokines, particularly interleukin-12 (IL-12) and tumor necrosis factor (TNF), which activate natural killer (NK) cells and neutrophils. Interferon-γ (IFN-γ) secreted by these cells triggers the production of microbicidal molecules (e.g., reactive oxygen species, ROS, and nitrogen species, NO), which are an essential component of the early response required to reduce parasite burden and initiate the adaptive immune responses specific to antigens necessary to control infection. B cells are responsible for the production of antibodies that promote the lysis of extracellular parasites or favor the phagocytosis of opsonized parasites, which is crucial in the case of *T. cruzi* infection, but can be less relevant in leishmaniasis, for example [39,50,51,52]. Despite an important role for humoral immune responses, as all the diseases discussed herein are caused by intracellular pathogens, parasite elimination depends on efficient antigen processing and presentation, leading to the involvement of T-cell-mediated immunity polarization directed to a T helper 1 (Th1)-type response. In this context, CD4^+^ T-cells play a central role in orchestrating macrophage activation and cytokine production, whereas CD8^+^ T cells contribute directly to intracellular parasite elimination through cytotoxic mechanisms. In contrast, regulatory T cells are required to further modulate this response and limit immunopathology [53,54]. Nonetheless, the immune response required to eliminate protozoan infections is challenged by parasite-driven immune evasion strategies, such as upregulation of IL-10, which drives the immune response towards a Th2 type, compromising the development of robust protective immunity [54,55,56,57].

Vaccination remains one of the most effective approaches for eliciting long-term protection against infections and to reduce the overall burden of parasitic diseases [58], current discussions have also focused on defining the most effective vaccination strategy—prophylactic or therapeutic. Prophylactic vaccines aim to prevent infection by inducing long-term immune memory, whereas therapeutic vaccines seek to control pathogen persistence and limit disease progression in infected individuals. For the majority of the protozoan diseases discussed here, the development of an effective prophylactic vaccine is essential to prevent infection and reduce transmission mainly in endemic areas [59,60,61]. In contrast, due to the low frequency of new infections and the high prevalence of chronic infection, together with the limited efficacy of current chemotherapy, an effective CD vaccine should ideally have a therapeutic profile [62,63]. Despite multiple attempts to generate effective vaccines for these four NTDs, the vast majority remain in the preclinical stage and only very few progress to human clinical trials. This may be due to limited reproducibility from animal to human models, as well as to the fact that clinical outcomes of these diseases depend on multiple factors, including the route of infection, parasite genetic diversity, and host immune competence, all of which influence infectivity levels and parasite resistance [62]. One major challenge for vaccine development for some of these four diseases is the extensive genetic diversity within parasite species, especially strains or lineages with distinct virulence profiles and immune evasion mechanisms. As a final challenge, vaccine strategies must also be capable of overcoming parasite-driven immune evasion mechanisms [56,64].

**Figure 2:**
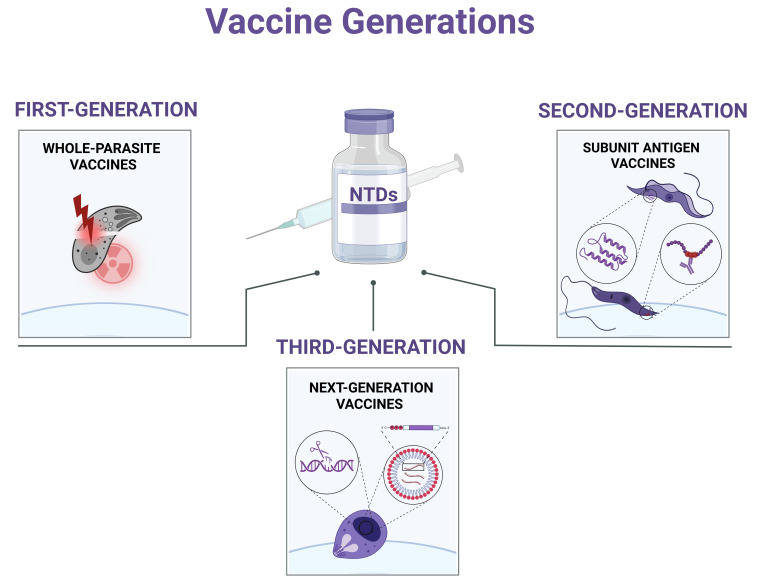
Types of vaccines for protozoan diseases. First-generation vaccines are based on whole parasites, either naturally attenuated or inactivated; second-generation vaccines are based on subunit antigens; and third-generation vaccines rely on genetic platforms, such as DNA, mRNA, or viral vectors, enabling in situ antigen expression, and include genetically modified live-attenuated parasites (GMLAP). NTDs, neglected tropical diseases. Created in BioRender. Brasil, M.E.C.G. & de Almeida-Júnior, C.R. (2026). https://BioRender.com/ly7jhue.

As for most vaccines, protozoan parasite vaccines can be classified into three groups, as summarized in Figure 2. First-generation vaccines included whole-parasite immunization using naturally attenuated strains or inactivated parasites (e.g., heat-killed, UV-irradiated, in vitro-attenuated organisms, or parasite lysates) [65,66,67]. Although naturally attenuated strains provide the complete parasite antigen repertoire, the potential for virulence reversion remains a major limitation. Second- and third-generation vaccines comprise immunizers based on subunit antigens (e.g., recombinant protein or RNA vaccines). These are both safer options but depend on rational antigen selection, as limited antigenic diversity can compromise immunogenicity and restrict cross-species or strain-specific protection [60,61,68]. For vaccines based on recombinant proteins, there is still a need to define adjuvants to boost the immune response. Using bioinformatics tools, epitope prediction enables a better antigen design, including multi-epitope and chimeric antigens, in which a combination of strong B- and T-cell epitopes may favor a balanced cellular and antibody-mediated immune response [69,70]. Using the same strategy, RNA-based vaccines can incorporate multiple sequences encoding antigenic targets. Despite clear preclinical advances using multiepitope technology, no immunizers based on the latter approaches have yet been approved for human use [69,71,72]. The only two human-licensed vaccines against parasitic diseases are based on a single antigen recombinant protein, and both are directed against malaria, as previously mentioned [8].

**Figure 3:**
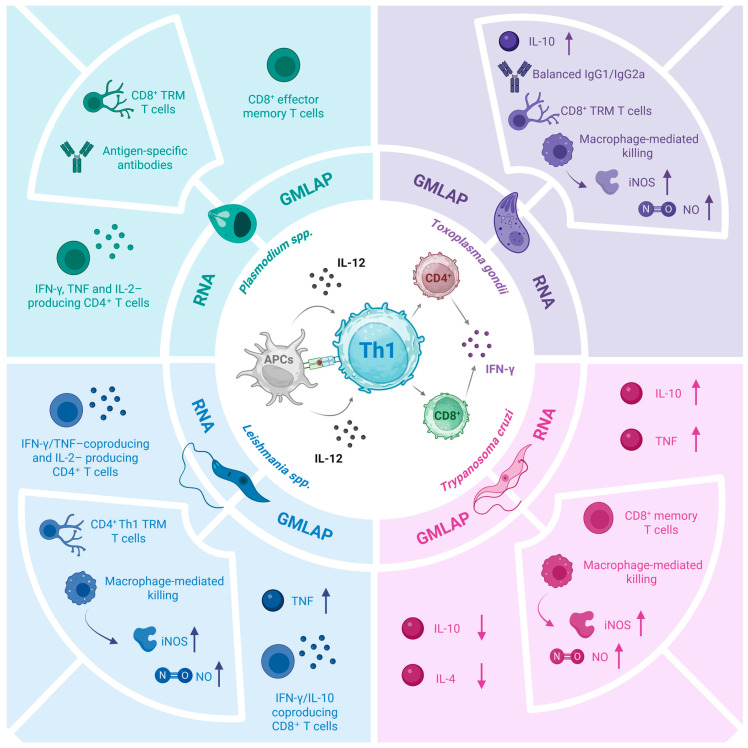
Immune responses induced by GMLAP and mRNA vaccines against intracellular protozoans. Immunization with each platform promotes APC activation, IL-12 production, and differentiation of CD4^+^ Th1 and CD8^+^ T-cells, leading to a robust IFN-γ production. This immunological axis underpins key effector mechanisms, including macrophage activation with iNOS expression and NO production, as well as TRM establishment, whereas specific differences depend on parasite biology and vaccine platform. ↑ indicates increase; ↓ indicates reduction. APCs, antigen-presenting cells; IL, interleukin; TNF, tumor necrosis factor; IFN-γ, interferon gamma; iNOS, inducible nitric oxide synthase; NO, nitric oxide; TRM, tissue-resident memory T cells; Th1, T helper 1; GMLAP, genetically modified live-attenuated parasite; IgG, immunoglobulin G. Created in BioRender. Brasil, M.E.C.G.& de Almeida-Júnior, C.R. (2026). https://BioRender.com/xax2l8u.

Data from the WHO and ClinicalTrials.gov indicate that approximately 142 malaria vaccine candidates have entered clinical evaluation over time, 104 of them were discontinued, and 38 remain active, including 17 that are still in the early stages of clinical development. Most candidates are based on recombinant subunit platforms, followed by virus-like particle (VLP), whole-parasite, RNA, recombinant viral vector, and self-assembling nanoparticle platforms, and predominantly target *P. falciparum* antigens [73,74]. In contrast, only five Leishmaniasis vaccine candidates have reached clinical trials, including recombinant protein vaccines such as Leish-111 (NCT00111553), LEISH-F2 (NCT01011309), and LEISH-F3 (NCT01484548), whole-parasite formulations such as autoclaved *L. major* (ALM) combined with alum (Alum-ALM) (NCT00429780, NCT00429715), and viral vectors such as ChAd63-KH (NCT03969134). Currently, only ChAd63-KH, a multi-epitope formulation combining the KMP-11 and hydrophilic acylated surface protein B1 (HASPB) antigens delivered by a chimpanzee-derived adenoviral vector, ChAd63, which in previous studies was demonstrated to induce immunogenicity through engagement of CD8^+^T-cells [59], remains under clinical investigation and has recently completed phase IIb, whereas most trials have been discontinued or continue to be investigated in canine models [75]. Notably, the ChAd63 viral vector has also been explored in malaria vaccine candidates such as ChAd63–MVA *P. vivax* Duffy binding protein (DBP) (NCT04009096) [74]. As shown in Figure 3, no vaccines being developed for toxoplasmosis and CD have yet reached clinical trials.

With the advent of clustered regularly interspaced short palindromic repeats-associated protein 9 (CRISPR/Cas9) technology in 2012 and RNA vaccine technology in 2020, preclinical studies using genetically modified live-attenuated parasite (GMLAP) vaccine candidates as well as RNA vaccines have become increasingly abundant, representing promising alternatives to improve vaccine efficacy [76,77]. As shown in Table 1, the protective immune responses elicited by GMLAP- and RNA-based vaccines are mostly dependent on a robust T-cell-mediated immunity, which is essential for controlling infections caused by the four intracellular parasites discussed here [78]. In the following sections, we review these two emerging strategies, GMLAP- and RNA-based vaccines, and illustrate where we stand in the process of developing new vaccines to control malaria, toxoplasmosis, leishmaniasis, and CD.

## 2. Genetically Modified Live-Attenuated Parasites: An Emerging Vaccine Platform

The biology of protozoan parasites and the diseases they cause have been extensively studied in the past few years using genetic manipulation techniques to interrogate gene function. In addition to facilitating functional characterization of genes involved in distinct stages of the protozoan life cycle, including virulence determinants, gene knockout strategies have contributed to the identification of promising new vaccine targets and to the development of novel vaccine platforms based on GMLAPs.

Until 10 years ago, gene knockout studies in protozoan parasites relied mainly on gene replacement using linear drug-resistance cassettes flanked by homologous gene arms, inserted into the parasite genome via homologous recombination (HR). The advent of CRISPR/Cas9 technology [79,80] had an enormous impact on studies involving gene manipulation in protozoan parasites, which were highly challenging due to the genomic complexity of these microorganisms [81,82,83,84]. In addition to facilitating basic studies, the CRISPR/Cas9 technology helped increase the efficiency of generating GMLAPs. Due to the broad presentation of antigens, GMLAP-based vaccines are highly promising because they mimic natural infection and induce robust protective immune responses that engage both humoral and T-cell-mediated immunity.

Despite their high efficacy, similar to studies using naturally attenuated parasite strains, GMLAP-based immunization strategies raise major safety concerns regarding potential reversion to virulence. These concerns can be mitigated through precise and controlled genetic engineering approaches, such as those enabled by the CRISPR/Cas9 system. Targeting multiple parasite genes as well as genes whose function cannot be restored through compensatory mutations is recommended, which might re-establish the virulent phenotype in GMLAP vaccines. Importantly, CRISPR/Cas9-based strategies allow the generation of non-transgenic parasites, since genome editing could occur without the requirement of inserting permanent drug-selection markers [77,81,85]. Further implications of using GMLAPs as a vaccine strategy include persistence of risk within the host, particularly in immunodeficient individuals. Here we discuss advances in preclinical and clinical studies, summarized in Supplementary Table S1, which evaluate GMLAPs as vaccine candidates for malaria, leishmaniasis, toxoplasmosis, and CD. Preclinical development is more advanced for *Plasmodium* and *Leishmania* spp., for which vaccine candidates have progressed to clinical evaluation or have reached the stage of vaccine production under Good Laboratory Practice (GLP) conditions.

### 2.1. Malaria

Among all four NTDs, only malaria has advanced GMLAP vaccines to clinical trials. Many years ago, tests with whole parasites as malaria vaccines, using radiation-attenuated *P. berghei* and *P. falciparum* sporozoites, resulted in partial protective immunity [86,87]. Chemoprophylaxis-assisted vaccination also resulted in partial protective immunity, although adverse effects were observed [88]. These early studies identified the pre-erythrocytic stage as a promising vaccine target since parasite numbers are still low at this point [89] and immunization with live parasites arrested during the liver stage (LS) was shown to induce sterile protection. Protection was mediated by IFN-γ produced by CD8^+^ T ells, supported by liver-resident memory T cells and antibody-mediated responses [90,91]. Although often resulting in incomplete attenuation and sporadic blood-stage (BS) breakthrough infections [92,93], initial studies have focused on single-gene knockouts. These studies laid the conceptual and experimental foundation by identifying possible targets and rational pathways for multigenic knockouts, providing more safety by complete attenuation, without compromising immunogenicity [94]. Based on a multigenic GMLAP strategy, the double-gene knockout parasite with genes encoding a micronemal 6-cysteine domain protein (*b9*), involved in hepatocyte invasion [95], and the sporozoite and LS asparagine-rich protein (*SLARP*), involved in transcriptional regulation [96], resulted in long-lasting sterile protection in mice. Preclinical data showed that *P. berghei* Δb9Δslarp sporozoites were arrested early in the LS and did not show a breakthrough in BS even in higher doses. The results of the clinical trials of candidate *P. falciparum* SPZ-GA1 demonstrated them to be well tolerated and also did not show a breakthrough in BS in malaria-naive adults ï. Of 25 volunteers, 3 developed sterile protection after controlled human malaria infection (CHMI) with wild-type *P. falciparum*, and most showed delayed parasitemia. The volunteers also had an increase in humoral and cellular immune responses, including anti-Pf circumsporozoite protein (CSP) antibodies and IFN-γ produced by CD4^+^ and CD8^+^ T-cells [96,97].

A triple-gene knockout vaccine candidate targeting pre-erythrocytic protein 52 (*p52*) and pre-erythrocytic protein 36 (*p36*), two micronemal proteins related to hepatocyte invasion and formation of the parasitophorous vacuole [98], and SLARP showed robust protection in mice for up to six months, with no BS breakthrough even after higher doses of challenge in susceptible mouse models. In clinical trials, *P. yoelii* *Δp52Δp36Δslarp* sporozoites were shown to be well tolerated in malaria-naïve adults without BS breakthrough. Immunized volunteers developed functional anti-PfCSP IgG and sterile protection against CHMI [76,99]. Both candidates, *P. berghei* *Δb9Δslarp* and *P. yoelii* *Δp52Δp36Δslarp*, are considered early-arresting GMLAPs (EA-GMLAPs) due to blocking of parasite development soon after hepatocyte invasion and showed a safe profile. Other multigenic strategies have focused on knockout parasites that are able to develop inside hepatocytes and arrest later during the LS (LARC-GMLAPs), aiming to increase antigen exposure to generate stronger protective immunity [76]. The double-gene knockout of stearoyl-CoA Δ9-desaturase (SCD), an endoplasmic reticulum enzyme responsible for the synthesis of oleic acid, and sporozoite conserved orthologous transcript 1 (SCOT1), a micronemal protein essential in LS development, related to apicoplast biogenesis, were arrested in the late LS due to defective apicoplast formation [100]. *P. berghei* *ΔscdΔscot1* sporozoites showed no BS breakthrough and, because of intrahepatic development, induced more robust CD8^+^ T-cell responses, as well as antibody production. Preclinical studies in mice also showed that the *P. berghei* ΔscdΔscot1 vaccine exhibits boost-dependent long-lasting sterile protection and is stage-transcending [101]. Double-gene knockout strategies also targeting genes essential for late LS development, such as meiosis inhibited-2–like (*PLASMEI2*), an RNA-binding protein [102], combined with liver-specific protein 2 (*LISP2*), a putative secreted protein during LS schizogony [103], or liver-stage nuclear protein (*LINUP*) [104], generated parasites that are arrested in the late LS without BS breakthrough. Vaccination with both *P. yoelii* *Δplasmei2Δlisp2* and *Δplasmei2ΔLINUP* (the second to be developed in *P. yoelii* and later translated to *P. falciparum*, named LARC2) induced antibodies against both LS and BS antigens, implying stage-transcending immunity, elicited robust and long-lasting protection, characterized by increases in liver-resident CD8^+^ T-cells and effector memory T-cell responses, as well as species-transcending protection, marked by heterologous challenge [93,104]. For safety reasons, blood-stage GMLAPs remain restricted to preclinical development, although mutants such as knob-associated histidine-rich protein (*KAHRP*)-deficient *P. falciparum* have been evaluated in humans in a dose-escalation study evaluating safety, infectivity, and immunogenicity [105]. Together, these studies showed that the combination of multiple gene deletions in GMLAPs is a promising strategy for next-generation malaria vaccines. A major challenge remains since most GMLAP platforms have been developed and tested in rodent malaria models, such as *P. berghei* and *P. yoelii*, and results obtained with these models of infection do not always translate directly to the five *Plasmodium* species that infect humans [106]. The difficulty of culturing and genetically manipulating *P. vivax* in the laboratory, combined with the formation of dormant hypnozoites during infection with this species, represents additional obstacles to experimental progress towards developing *P. vivax* GMLAPs [106,107]. Overcoming all these limitations is essential to fully explore the broader potential of GMLAP-based malaria vaccines.

### 2.2. Toxoplasmosis

The S48 strain-based formulation Toxovax^®^ is a live-attenuated vaccine and the only product commercially licensed for toxoplasmosis, but approved for veterinary use only, more specifically, to prevent congenital toxoplasmosis in sheep [108]. It is not a GMLAP, since the strain used became attenuated through consecutive and prolonged passages in mice over many years, a process that resulted in the loss of its capacity to form tissue cysts and oocysts [108,109]. Similar to Toxovax^®^, other attenuated *T. gondii* strains have been generated using irradiation and chemical exposure that resulted in reduced virulence and a potential vaccine profile [110].

Due to its haploid genome, genetic manipulation in *T. gondii* is facilitated in a way that a deletion of a gene requires targeting only a single allele [111]. Using CRISPR-Cas9 strategies, several *T. gondii* target genes encoding proteins involved in parasite invasion, intracellular survival, and host–parasite interaction, particularly those associated with secretory organelles, have been generated and tested as vaccine candidates [112]. These studies have targeted dense granule protein genes, such as *Gra76*, *Gra72*, and *Gra5*, which are involved in parasitophorous vacuole remodeling and cyst formation [113]; proteins containing C2 membrane-binding domains, associated with vesicular fusion and transport events for invasion, organelle secretion and host–cell interaction [114]; and metabolic enzymes, such as 6-phosphogluconate dehydrogenase 1 (6PGDH1), a key component of the pentose phosphate pathway [115] and regulatory phosphatases like the catalytic subunit of protein phosphatase 2A (PP2A-C) involved in signaling cascades that control parasite replication and invasion [116]. Mice immunization with these mutants resulted in a Th1-biased immune response [117], but most studies showed only partial protection against acute and chronic infection, even when evaluated in feline models as well.

Among the *T. gondii* GMLAP candidates, mutants that target rhoptry-associated protein genes such as *ROP18* and *ROP38*, kinase-related genes such as *CDPK3*, and genes associated with metabolic pathways, such as the double knockout of *Ompdc* and *Uprt* genes, stand out as well-characterized live-attenuated vaccinal strains [118]. Mutant knockouts of *ROP18* in the WH3 strain (WH3Δrop18) and *ROP38* in the Pru strain (Pru∆rop38) exhibit reduced virulence and can induce total protection against highly virulent Type I RH as well as Type II and Chinese 1 genotype strains (WH3, WH6) [119,120]. Immunization with these mutants triggers strong cellular and humoral responses, with an increase in IFN-γ, IL-12, TNF, and levels of IgG2a, leading to decreased brain cyst formation and prolonged survival after lethal challenge. In addition to cytokine production, vaccination with these mutants was associated with activation of CD4^+^ and CD8^+^ T-cells, indicating a central role for cellular immunity in protection. These mutants can provide short- and long-term efficacy against acute and chronic toxoplasmosis in mice and significantly reduce the parasite burden in the brain following challenges with different strains. The same pattern of cellular, cytokine, and humoral response was observed and well characterized in the deletion of a gene that encodes Ca^2+^-dependent protein kinase 3 (CDPK3) in a ME49 strain (ME49Δcdpk3). *T. gondii* egress from infected cells is regulated by intracellular calcium signaling, which can be controlled by these calcium-dependent protein kinases. Immunization with *ME49Δcdpk3* resulted in effective protection against several different types of strains, RH, ME49, WH3, and WH6, and a significant reduction in cyst formation [121]. It is noteworthy that all of these three knockout mutants were able to induce a Th1 response while also inducing levels of IL-10, an essential regulatory cytokine that helps avoid tissue damage from excessive inflammatory responses during infection [119,122].

Another advanced strategy is the double-knockout mutant targeting orotidine-5 monophosphate decarboxylase (*ompdc*) and uracil phosphoribosyltransferase (*uprt*) genes, which was generated in RH strains (*RHΔompdcΔuprt*) [123]; both genes are involved in pyrimidine biosynthesis. The double-knockout mutant is incapable of sustained cellular replication in human foreskin fibroblasts (HFFs), but it is capable of inducing robust protective immunity against acute and chronic toxoplasmosis in both models [115]. Protection after inoculation of this mutant was evaluated in murine models challenged with tachyzoites of different strains of *T. gondii*, as well as in felines challenged with ME49 tissue cysts, showing a significant reduction in oocyst shedding in cats. This is particularly relevant because cats are the definitive hosts of *T. gondii* and can shed environmentally resistant oocysts, thus playing a central role in parasite transmission and environmental contamination [124]. Furthermore, RHΔompdcΔuprt was further evaluated through passive immunization assays and showed that immunized sera and splenocytes, especially CD8^+^ T-cells, significantly extended mice survival after challenge compared with naive mice. Nevertheless, despite these promising preclinical findings, these vaccine candidates still need to satisfy key regulatory requirements before advancing to clinical translation, including Good Manufacturing Practices (GMP) production and standardized immunization protocols.

### 2.3. Leishmaniasis

Leishmanization, which consists of the intradermal inoculation of live *Leishmania major* that results in a spontaneous healing skin lesion, was widely practiced in the Middle East for many years and provided strong evidence for the efficacy of the vaccine strategy using live parasites. In humans, controlled primary infection with live *L. major* induces robust, naturally acquired protective immunity, leading to a reduced incidence of the disease and resistance to reinfection. However, the practice was discontinued due to safety and ethical concerns, since non-healing skin lesions and exacerbation of skin diseases may occur [125]. Genetic manipulation techniques, which became available for different *Leishmania* species in the early 1990s, allowed the development of GMLAPs that have an attenuated phenotype determined by the deletion of virulence genes in the parasite genome. Mouse immunization with the first knockout mutant, the *L. major* dihydrofolate reductase–thymidylate synthase (DHFR-TS) [126], conferred cross-protection against *L. amazonensis* [127]. This work laid the foundation for targeted gene disruption across *Leishmania* spp. and other trypanosomatid parasites, facilitating the rational design of GMLAPs to be used as a vaccine.

GMLAP vaccine strategy against VL has been explored in many preclinical studies, such as the ones targeting metabolic pathway-related genes, including *Hel67* a nucleolar RNA helicase involved in rRNA processing [128], and arabino-1,4-lactone oxidase (*ALO*) [129], which codes an enzyme that catalyzes the final step in ascorbate biosynthesis [130]. Other studies explored the heat shock protein 70-II (HSP70-II), a molecular chaperone [131,132,133,134]. These studies showed promising protective mechanisms through a Th1-biased immune response, promoting humoral responses and limiting disease severity in mice and hamsters. Although rodent models, including mice and hamsters, are useful for initial studies of *Leishmania* infection, variability in parasite species and host genetics, along with inconsistent results regarding visceralization, limits their predictive value for VL in these preclinical tests.

Recent studies with *p27* and *centrin-1* null mutants, which have advanced further in preclinical development, showed promising results in both rodent models and dogs. *p27* encodes a subunit of the cytochrome c oxidase complex of the mitochondrial membrane, which results in reduced ATP production in *p27* knockout intracellular amastigotes [135]. Among all *Leishmania* GMLAP vaccine candidates, centrin-1 is the most extensively studied, with the generation of knockouts in different *Leishmania* spp. Centrin-1 is a basal body calcium-binding protein essential for organelle duplication and cell cycle progression in *Leishmania* [136]. Immunization with *p27* or *centrin-1* null mutants conferred similar protection characterized by NO-dependent mechanisms that contribute to the reduction in parasite burden and disease progression. Long-term protection is associated with humoral responses along with the activation of multifunctional CD4^+^ and CD8^+^ T-cells, including IFN-γ/IL-10 co-producing populations, generating a balanced Th1/Th2 response [137,138,139,140,141,142]. This phenomenon had previously been observed during protection in mice immunized with HSP70-II^−/−^ parasites for both CL or VL using a murine model [122,123]. A balanced Th1/Th2 response is critical for limiting disease-associated organ damage, as IL-10 plays a central role in restraining infection-induced immunopathology [135,136]. In human CL, it is known that excessive IFN-γ-driven inflammation correlates with lesion severity, whereas regulated IFN-γ/IL-10 production mitigates tissue injury but may allow some degree of parasite persistence [143,144]. A similar immunoregulatory balance has also been described in VL patients, with IL-10 helping limit immunopathology [145].

*p27* and *centrin-1* null mutants represent promising GMLAP candidates, as they can confer cross-protection against clinically relevant species, including *L. infantum* and *L. major*, and have their safety and efficacy also demonstrated in dogs, with significant protection and reduced organ damage [146,147]. Further advances have been achieved with centrin-1 mutants thanks to the use of CRISPR/Cas9-based genome editing, which allowed the generation of genetically modified parasites without antibiotic markers [77,147]. A recent study with mutants of the GPI anchor biosynthesis cofactor PBN1 (GPI-MT) demonstrated reduced parasite burden in the visceral organs of vaccinated mice [148]. Despite these advances, great challenges are still present down the road towards a commercially available GMLAP product to control leishmaniasis, one of which is large-scale production under GMP. Centrin-1–based GMLAP vaccines are the only product that has been successfully produced on a large scale and under GLP conditions. Preclinical toxicity studies in hamsters showed adverse effects after subcutaneous administration of 9 million/dose/animal. Importantly, freeze–thaw conditions did not affect parasite viability, being stable at 4 °C for at least 6 h, supporting their potential use in clinical trials and eventual commercialization [149].

### 2.4. Chagas Disease

Compared with the other three NTDs discussed here, fewer advances have been made using GMLAPs to develop a vaccine for CD. Early approaches aimed at generating attenuated strains that could provide strong protective immune responses focused on targeted gene deletion in naturally attenuated *T. cruzi* isolates using conventional gene knockout protocols. Monoallelic disruption of *DHFR-TS* and calreticulin (*TcCRT*) in the low-virulence TCC strain resulted in reduced infectivity in mice but did not confer greater protection compared to the wild-type strain [150,151]. Deletion of genes involved in metabolic pathways in the CL strain, specifically enoyl-coenzyme A (CoA) hydratase 1 and 2 (*ECH1/ECH2*), resulted in a mutant parasite that was tested as an oral vaccine, but did not enhance responses mediated by CD8^+^ T-cells [141]. A more successful result was obtained with the CL Brener strain, with deletion of the *lyt1* gene, which encodes a surface protein that is a key factor in *T. cruzi* infectivity and pathogenesis. Inoculation of the null mutant lyt1 induced persistent attenuated infection with minimal tissue damage and elicited *T. cruzi*-specific antibodies in vaccinated mice. Although post-challenge histopathology was not assessed, it was shown to fully protect immunized mice with no detectable parasitemia [152].

A hallmark of chronic cardiac disease (CCD) in patients with CD is elevated plasma concentrations of inflammatory biomarkers, including IL-6, IFN-γ, TNF, CXCL-10, and CCL-5, associated with the severity of cardiomyopathy. In contrast, IL-17 (Th17 response) production has been associated with reduced cardiac damage. Moreover, sustained IFN-γ production and a balanced TNF/IL-10 ratio have been linked to the development of digestive clinical manifestations, whereas IL-10 production may serve as a crucial biomarker during the indeterminate phase of the disease [153,154]. Since in chronically infected CD patients, exacerbated inflammation is responsible for the pathology, when considering the use of GMLAPs as a vaccine candidate, it is essential that immunization induces a balanced Th1-mediated response, which is sufficient to eliminate the intracellular parasite without causing tissue damage. From this perspective, a preclinical study demonstrating that gene deletion of the cyclophilin-19 (*Cyp19*) gene in the Brazil strain constitutes a promising vaccination strategy capable of improving CD8^+^ T-cell response without causing tissue injury represents a significant advancement [155]. Cyp19 is a secreted epimastigote peptidyl-prolyl cis–trans isomerase (PPIase) involved in neutralizing proline-rich insect antimicrobial peptides [156]. Mice vaccinated with the *Cyp19* knockout strain exhibited robust IFN-γ production, indicative of a Th1-biased immune response, along with reduced levels of IL-4 and IL-10 and activation of B-cell responses, including trypanolytic antibody production. The ability of *Cyp19* mutants to infect host cells without sustained amastigote multiplication results in intracellular antigen presentation and a strong T-cell-mediated immune response. Accordingly, no detectable amastigote nests were observed in the organs of immunized mice, nor were parasites detected in the bloodstream during long-term follow-up. Importantly, no tissue damage was observed in vaccinated and posteriorly challenged mice, resulting in full protection [155].

One distinctive feature of the *T. cruzi* genome is the presence of multicopy gene families, several of them encoding virulence factors [157]. Functional genomic studies aimed at characterizing the role of these genes have been hindered not only due to the large number of repetitive sequences but also to increased parasite genetic complexity due to the existence of strains with hybrid genomes in which two alleles of the same gene can be highly divergent. Only after the advent of CRISPR/Cas9 technology did it become possible to generate knockout parasites targeting multigenic families [82,158]. Surface *Trans*-sialidases (TS) are encoded by the largest multigenic family present in the *T. cruzi* genome with over 1400 gene copies, but only 12–16 gene copies encode enzymatically active enzymes (aTS) [159]. aTS mediates the transfer of sialic acid from host glycoconjugates to parasite surface mucins, thereby promoting immune evasion [160] and weakening host cellular responses by re-sialylating immune receptors such as CD43 on CD8^+^ T-cells [161]. Disruption of 16 *aTS* copies, using CRISPR/Cas9, in the CL Brener genome resulted in the generation of a highly attenuated mutant with an impaired capacity to establish infection even in the immunodeficient IFN-γ knockout mice [159]. Preclinical studies showed that mice immunized with a single dose of the aTS mutants and subsequently challenged with the virulent Y strain were fully protected, exhibiting undetectable parasitemia and no detectable tissue parasitism, as assessed by PCR and bioluminescence. This protection is mediated by both humoral and a Th1-biased immune response characterized by IFN-γ production [159]. In vitro infection studies showed that, although aTS null mutants were able to invade cells and replicate as amastigotes, they had impaired ability to differentiate from amastigotes to trypomastigotes and thus did not propagate the infection. Although aTS mutants cannot complete the lytic cycle in the infected cell, they elicit a highly protective immune response. Although further characterization of this strain to be used as a GMLAP vaccine is still necessary because multiple genes have been disrupted, it is considered a promising candidate for testing as a canine vaccine.

One reason that may explain the limited progress in the development of *T. cruzi*-based GMLAP vaccines is the fact that genome assembly in this parasite is still highly challenging because of the enormous repetitive content that is a hallmark of the *T. cruzi* genome [157]. Notably, only in 2026, long-read sequencing technologies enabled the generation of a telomere-to-telomere assembly of the Dm28c strain genome [162]. Together with advances in gene editing technologies, this represents a milestone that is expected to reshape future studies on vaccine development. A second major reason is the fact that, among NTDs, CD remains one of the least funded diseases worldwide, receiving only 0.8% of total funding compared with approximately 17.4% for malaria, which has contributed to slower progress in advancing both vaccine platforms [163].

In addition to requiring better characterization of the attenuated strains, including addressing safety questions, the use of GMLPs as vaccine candidates against CD also requires additional preclinical data to provide a better understanding of disease progression in vaccinated and control animals, particularly in animal models for chronic infection. Therapeutic vaccines are considered the preferred approach to CD as they can help contain tissue damage associated with chronic *T. cruzi* infection if administered during the early stages of the chronic phase. In addition, if associated with drug treatment, they may reduce the drug concentration required to control infection with low toxicity. Increased efforts are also necessary towards improving the characterization of animal models for chronic *T. cruzi* infection, including mice and dogs, as well as towards the production of clinical batches of *T. cruzi* GMLAPs [62].

## 3. mRNA-Based Vaccine Strategies Against Parasitic Infections

In light of recent advances in vaccine technology that unveiled the potential of messenger RNA (mRNA)-based platforms as a versatile and effective strategy for controlling infectious diseases during the COVID-19 pandemic [78], many efforts have been devoted to the development of mRNA vaccine formulations with sequences from *Plasmodium*, *Leishmania*, *Toxoplasma*, and *Trypanosoma* antigens. mRNA vaccines offer several advantages over traditional platforms, including their capacity to express complex antigens in their native conformation, rapid production timelines, and the ability to stimulate both humoral and cellular immune responses [164,165]. Because mRNA vaccines lead to antigen presentation through class I and II of the major histocompatibility complex (MHC) and elicit a robust Th1-polarized immune response characterized by IFN-γ production and activation of CD8^+^ T cells, mRNA vaccines are particularly useful for preventing intracellular parasite infections. Thanks to advances in lipid nanoparticle (LNP) delivery systems and nucleoside modification technologies, current protocols have improved mRNA stability, translational efficiency, and immunogenicity, while avoiding excessive innate immune response activation [166].

Conventional mRNA, circular RNA, and self-amplifying RNA (saRNA) vaccines share the common principle of delivering RNA molecules encoding antigenic proteins for intracellular expression, but differ in structure, delivery, and optimization strategies. Conventional mRNA and circular RNA encode only the antigen and typically require LNP formulations to ensure RNA stability and efficient cellular uptake, whereas saRNA contains additional replicase genes derived from alphavirus replicons that enable intracellular RNA amplification and prolonged antigen expression with lower RNA doses [167]. Most studies on infectious-disease mRNA vaccines have focused on conventional mRNA platforms and LNP formulations using different lipid compositions [168]. Besides defining the coding sequence for the antigen, the design of these vaccines involves testing different 5′ and 3′ untranslated sequences (5′ and 3′ UTRs), the presence of signal peptide to promote secretion of the translated antigen or signals for glycosylphosphatidylinositol (GPI) anchor addition as well as optimization of several parameters that will impact translation efficiency including protocols to reduce double-strand RNA (dsRNA) contaminants [78].

Besides COVID-19 vaccines, the only mRNA vaccine currently approved and commercialized for an infectious disease is mRESVIA, designed to prevent respiratory syncytial virus (RSV) infection in older adults. Phase III clinical trials showed that this vaccine, formulated in LNPs containing mRNA encoding the stabilized prefusion form of the RSV F glycoprotein, elicits strong neutralizing antibody responses and substantial protection against RSV-associated lower respiratory tract disease [169]. Although several other mRNA vaccines targeting infectious diseases are being evaluated in clinical trials, including mRNA vaccines against influenza, cytomegalovirus (CMV), Zika virus, and HIV, as well as various therapeutic cancer vaccines, only one clinical trial of mRNA vaccines against a parasitic disease is currently underway. As shown in Supplementary Table S2, although several studies describing preclinical data on RNA-based vaccines against toxoplasmosis, leishmaniasis, and CD have been published since 2006, the prospects for clinical trials for any of these three diseases are still very limited.

### 3.1. Malaria

A first-in-human phase I study is evaluating a vaccine candidate that encodes the CSP of *P. falciparum*, the major surface antigen on sporozoites, the infectious form of *Plasmodium* transmitted by mosquito bites, that migrate to the liver to initiate infection and are key targets for neutralizing antibodies that block hepatocyte invasion [170]. CSP, the antigen component of the two approved recombinant malaria vaccines, has been broadly explored in mRNA vaccine studies using different *Plasmodium* species and animal models of infection [171,172,173,174,175]. Mice immunization with an mRNA vaccine encoding a modified CSP sequence from *P. berghei* (PbCSP), in which the signal peptide, repeat region, and GPI anchor were deleted from the wild-type CSP sequence, induced a potent CD8^+^ T-cell response that included memory T cells resident in CD8+ tissue. PbCSP RNA immunization resulted in strong protection against challenge, evidenced by reduced parasitemia and half of the mice exhibiting sterile protection. In another study, a formulation containing wild-type CSP RNA sequences from *P. vivax* resulted in robust cellular and humoral immune responses against CSP and its repetitive region. Using a challenge protocol based on transgenic sporozoites expressing *P. vivax* CSP, this study demonstrated that approximately 90% of sterile protection is achieved [175]. The CSP from *P. falciparum* was also tested as a malaria mRNA vaccine using a construct lacking the GPI anchor sequence and a challenge protocol also based on transgenic *P. berghei* parasite strains. This formulation resulted in strong humoral and cellular immune responses and provided 88% sterile protection after an immunization protocol that consisted of three doses administered at 6-week intervals [171].

To enhance immune responses induced by malaria mRNA vaccines, additional modifications in RNA sequences have also been explored. The antigen CSP of *P. falciparum* was fused to macrophage inflammatory protein-3 alpha (MIP3α), a chemokine that binds to the CCR6 receptor expressed on the surface of dendritic cells. The study showed that the mRNA platform induced high levels of antigen-specific antibodies, with even greater responses observed when the construct included MIP3α was compared with conventional mRNA. Although both conventional mRNA immunization and the MIP3α-fusion mRNA reduced liver-stage burden, the fusion construct provided enhanced protection, associated with increased antibody levels against the central repetitive NANP region of CSP. Furthermore, CSP MIP3α-fusion mRNA increased the percentage of CD4^+^ T cells expressing IFN-γ, TNF, and IL-2, as well as CD8^+^ T cells expressing IFN-γ. The same study also showed that gradual dose reduction and extending the interval between boosts from 2 to 6 weeks further improved protection. These findings highlight the importance of optimizing existing platforms to potentiate immune responses [172].

Furthermore, to improve the magnitude of the immune response, co-immunization with different *P. falciparum* mRNAs has been evaluated, as shown in a study in which CSP and Pfs25 mRNAs were combined. Pfs25 is a protein expressed on the surface of mosquito ookinetes and represents an important target for transmission-blocking vaccines (TBVs). However, despite inducing extremely potent immune responses and transmission-reducing antibody activity, the co-immunized group did not show superior responses compared with immunization with either of the two immunogens individually [173]. Nevertheless, since TBVs focus on parasite stages that develop in the mosquito, including gametes, zygotes, and ookinetes, antibodies induced by vaccination may affect parasite survival in the mosquito vector, thereby preventing new human infections [176]. The ookinete surface protein from *P. vivax* has also been tested (Pvs25), and the results showed that immunization with this mRNA induced high levels of parasite-specific antibodies with 100% transmission-reducing activity (TRA), indicating the serum’s capacity to block malaria parasite development in mosquitoes. In this study, the immune response was followed for 7 months to evaluate durability and demonstrated that although antibody levels declined over subsequent months, the mRNA/mRNA prime-boost regimen generated the highest responses, whereas the protein/protein regimen showed the lowest. In addition, TRA efficacy was >99% for the mRNA/mRNA vaccination protocol, compared with 63% for the protein/protein regimen [177].

Few other malaria targets have been explored using mRNA vaccine platforms. This is important since a combination of mRNA sequences that encode antigens expressed in LS, such as sporozoites and BS, or merozoites, is expected to produce the most efficacious results. Immunization of non-human primates with mRNA encoding the *P. falciparum* glutamic acid-rich protein (PfGARP), an antigen expressed on the surface of infected erythrocytes, elicited strong IgG antibody responses and significantly reduced parasitemia following challenge with red blood cells infected with blood-stage *P. falciparum* [178]. mRNA containing sequences encoding the secreted cell-traversal protein for ookinetes and sporozoites (CelTOS), a highly conserved protein involved in the invasion of hepatocytes in the vertebrate host and mosquito midgut cells, was also tested in a mouse model of infection. Despite inducing potent cellular and humoral responses after a three-dose immunization regimen, this formulation did not confer significant protection against challenge with a mosquito-bite inoculum of transgenic *P. berghei* expressing the *P. falciparum* antigen [179]. In contrast, immunization with mRNAs encoding the blood-stage *P. yoelii* merozoite surface proteins MSP1 and MSP8 showed significant protection in a mouse model of infection [180]. These authors also showed that mRNA immunization induced higher antibody titers against both vaccine components when compared with immunization with the corresponding recombinant protein. Following challenge with *P. yoelii*-infected red blood cells, 50% of immunized animals showed no detectable parasitized RBCs, with the mean peak parasitemia in mRNA-immunized mice significantly lower than that in the group that received the recombinant protein [180].

Using a distinct self-amplifying RNA platform, the effect of developing an immune response directed against Plasmodium macrophage migration inhibitory factor (PMIF), a parasite protein that modulates the host inflammatory response during liver-stage parasite development. In a mouse model of infection, the formulation encoding *P. berghei* MIF induced strong IgG responses, IFN-γ-producing CD4^+^ T cells, and liver-resident memory CD8^+^ T cells and conferred protection against both infection and reinfection, as evidenced by reduced parasitemia, prolonged mean survival time, absence of parasites in the spleen, and reduced liver parasite burden [181].

Other mRNA delivery systems have also been explored for the development of malaria vaccines, including lipoplex (LPX)-formulated mRNA encoding the large ribosomal subunit protein L6 (RPL6) of *P. berghei*. Immunization using the type I NKT cell agonist α-galactosylceramide (αGC), as an adjuvanted induced RPL6-specific TRM CD8^+^ T cells and increased the pool of circulating memory T cells in the spleen. Importantly, approximately 80% of vaccinated mice remained free of parasitemia, demonstrating sterile protection. Notably, the protective effect of RPL6 mRNA alone was limited. Another key finding is that prior parasite exposure did not impair the immune response to the adjuvanted mRNA vaccine. In contrast, pre-exposed mice vaccinated with attenuated sporozoites showed reduced T-cell responses, highlighting a potential advantage of mRNA vaccines in endemic settings [182].

### 3.2. Toxoplasmosis

One early study investigating RNA-based immunization against *T. gondii* employed total parasite-derived RNA administered intranasally to mice [183]. Mice immunized and subsequently challenged with a lethal dose of cysts from the 76K strain exhibited a 50% survival rate compared to non-immunized controls. Notably, immunized mice challenged with a sublethal dose of the same strain demonstrated a 32% reduction in cyst burden relative to the non-immunized group. More recent investigations have incorporated in vitro-transcribed mRNA and self-amplifying RNA platforms, utilizing advanced delivery systems to enhance cellular uptake, including LNPs and dendrimers. A self-amplifying RNA vaccine encoding *T. gondii* NTPase-II induced robust humoral and Th1-biased cellular responses, leading to prolonged survival and reduced parasite burden, particularly when delivered via LNPs, highlighting its potential against acute and chronic toxoplasmosis [184]. Immunization with self-amplifying RNA encoding multiple *T. gondii* antigens, encapsulated within dendrimer-based nanoparticles, also conferred complete protection against a lethal challenge in mice following a single-dose administration [185].

Another study investigating a multi-antigen vaccine platform targeted an antigen expressed across the distinct developmental stages of *T. gondii*. A quadrivalent self-amplifying mRNA-LNP vaccine encoding four *T. gondii* antigens elicited a robust humoral response and a Th1-skewed cellular immune profile, characterized by increased production of pro-inflammatory cytokines such as IFN-γ. Furthermore, mice immunized with this formulation exhibited prolonged survival in the acute-infection model following challenge with the type I RH, type II ME 49, and locally isolated WH6 strains, as well as a reduced cyst burden in the chronic infection model [186]. Finally, a series of studies demonstrated that immunization with LNP-formulated mRNA encoding antigens selected through epitope prediction (SAG1 and TG290) elicited robust IgG responses characterized by Th1-biased features, enhanced CD4^+^ and CD8^+^ T-cell activation, and increased IFN-γ production, ultimately resulting in significantly prolonged survival following lethal challenge with the RH strain [187,188,189].

### 3.3. Leishmaniasis

Only three published studies have evaluated mRNA-based immunization strategies for the development of vaccines against leishmaniasis. The immune response in animals immunized with mRNA formulations containing sequences of the *L. infantum* kinetoplast-associated antigen named LinKAP, a conserved *Leishmania* protein that is highly immunogenic, was compared with the response generated by immunization with the corresponding recombinant protein. It was demonstrated that although the mRNA formulation induced higher levels of antibodies in immunized mice compared with mice immunized with the recombinant LinKAP protein, no significant differences in IFN-γ production were observed in mice immunized with the LNP-mRNA or the recombinant protein formulations [190].

In a model of cutaneous leishmaniasis, an mRNA vaccine encoding phosphoenolpyruvate carboxykinase (PEPCK) was shown to induce robust T-cell responses and expansion of Th1 and T follicular helper (Tfh) cell populations, although no significant protection against *L. major* challenge was observed. When combined with IL-12 mRNA formulated in lipid nanoparticles, the vaccine promoted the expansion of tissue-resident memory T cells, which are important for rapid local responses and recruitment of other immune cells. This strategy resulted in protection characterized by reduced lesion size, lower pathological scores, and decreased parasite burden in the ear after L. major infection [191]. Using a heterologous vaccination strategy, in which RNA vaccine and recombinant protein were combined, the sequences encoding the recombinant fusion antigens LEISH-F2 and LEISH-F3 of *L. donovani*. were evaluated, the RNA formulation based on the self-amplifying replicons platform. This study showed that RNA priming followed by protein boosting induced intermediate levels of antigen-specific antibodies, strong CD4^+^ T-cells co-producing IFN-γ and TNF, and IL-2-producing CD4^+^ T-cells, resulting in improved protection against *L. donovani* challenge with reduced parasite burden in the liver [192].

### 3.4. Chagas Disease

mRNA-based vaccines have also been tested as a promising strategy for CD due to their capacity to elicit a Th1-polarized immune response and to promote the activation of cytotoxic T lymphocytes, but similar to toxoplasmosis and leishmaniasis, only a few published studies in animal models of CD have been described [168]. The immunogenicity of an mRNA-LNP vaccine encoding the *T. cruzi* flagellar protein antigen (Tc24) was evaluated in homologous and heterologous immunization protocols combined with the corresponding recombinant protein. A heterologous vaccination using Tc24 mRNA to prime and Tc24 protein to enhance polyfunctional CD8^+^ T-cell responses promoted Th1, Th2, and Th17-associated cytokine profiles, and induced stronger humoral immune responses [193]. Based on these findings, the efficacy of a bivalent mRNA-LNP vaccine encoding the antigens Tc24 and amastigote surface protein 2 (ASP-2) was evaluated in a chronic murine model of CD. The bivalent formulation showed a more sustained cytokine response than the monovalent formulations and reduced the parasite load, as well as attenuated cardiac inflammation [63].

The chaperone Tcj2 antigen, selected through immunopeptidomic analysis, was also tested as a target antigen in studies of CD mRNA vaccine due to the predicted capacity of this antigen to elicit cytotoxic T-lymphocyte responses. Immunization with Tcj2 mRNA formulated in LNPs elicited robust cytotoxic CD8^+^ memory T-cell responses, characterized by increased production of IFN-γ, TNF, granzyme B, and perforin, along with elevated IgG2c levels, consistent with a Th1-biased immune profile. Furthermore, splenocytes isolated from immunized mice were able to restrict *T. cruzi* replication in vitro, supporting Tcj2 as a promising target antigen for vaccine development [194].

A recent study from our group evaluated mRNA formulations encoding the *T. cruzi* Trans-sialidase (TS), a surface protein known to act as a powerful virulence factor, and compared their efficacy of protection with a vaccine based on the corresponding recombinant protein. As indicated above, TS is encoded by a multigene family, and only a few TS contain a repeat C-terminal amino acid domain known as Shed Acute Phase Antigen (SAPA), which is highly immunogenic but acts as part of the parasite’s immune diversion strategy. In addition to confirming the immunodominance of the SAPA domain, it was shown that immunization with the TS protein or RNA containing sequences with or without the repeat domain resulted in strong humoral and cellular responses. Although after challenging immunization of mice with *T. cruzi* trypomastigotes, the presence of the repeats did not significantly impact protection, and immunization with TS mRNA containing the SAPA domain resulted in a robust humoral response with a more balanced cellular immune profile characterized by the production of both IFN-γ and IL-10. In the study, both groups of vaccinated animals exhibited reduced parasitemia and decreased cardiac parasite burden, but, notably, elevated IL-10 levels in animals immunized with TS sequences containing the SAPA domain correlated with diminished inflammatory infiltrates in the hearts of immunized mice [68].

## 4. Perspectives, Limitations, Regulatory Challenges, and Heterologous Vaccine Regimens to Control NTDs

Although NTD control using GMLAP- and mRNA-based strategies clearly hold significant promise, advances in the development of new vaccines based on these two technologies have differed greatly when the diseases are compared. This is in part due to the intrinsic differences in complexity of the parasite and the disease they cause, as well as to great differences in the availability of research funding for both basic and applied studies involving these different pathogens.

In parallel with the increase in efficacy, the advancement of novel vaccine technologies has been accompanied by increasingly stringent regulatory and manufacturing requirements, particularly in terms of product characterization, quality control, consistency, and large-scale production, which are all essential steps in vaccine development aimed at reaching a commercial product.

Regarding the use of GMLAPs, regulatory guidelines require a thorough molecular characterization, the assessment of off-target effects, and consistency in product delivery. In addition, regulatory expectations are particularly stringent with respect to genetic stability, ensuring the absence of reversion to virulence, as well as controlling biodistribution and shedding profiles [195]. From a scalability perspective, the large-scale production of GMLAP-based vaccines requires controlled bioreactor systems to ensure reproducibility, product consistency, and compliance with regulatory standards, increasing overall production costs. Key regulatory considerations for mRNA vaccines include the physicochemical properties of lipid nanoparticles (e.g., size, polydispersity, and composition), the biodistribution of these particles across tissues, and critical attributes of the mRNA construct, such as sequence design, structural elements, and stability, all of which must be tightly controlled to ensure safety, efficacy, and manufacturing consistency. Key regulatory considerations for mRNA vaccines include the physicochemical properties of lipid nanoparticles (e.g., size, polydispersity, and composition) and critical attributes of the mRNA construct, such as sequence design, structural elements, and stability, all of which must be tightly controlled to ensure safety, efficacy, and manufacturing consistency [190]. Moreover, both GMLAP- and mRNA-based vaccines require ultra-low temperature storage to maintain batch stability, imposing the need for a robust cold chain during distribution [196]. However, for GMLAP-based vaccines, an additional challenge lies in ensuring the viability and consistency of live cells across batches [149]. This requirement poses significant challenges for vaccine deployment in remote and resource-limited settings with limited infrastructure, particularly considering that NTDs affect low-income countries that often rely on subsidies to access vaccines. The COVID-19 pandemic further highlighted these disparities, revealing pronounced inequities in vaccine access, especially in distribution across African countries [197].

As shown in the comparative overview that is summarized in Table 2, each of the three other vaccine platforms, i.e., vaccines based on recombinant protein, DNA plasmids, and viral vector vaccines, has its pros and cons. Considering the advantages and limitations of each vaccine platform, preclinical and clinical studies have been conducted towards vaccine development for the four NTDs discussed here with all five vaccine platforms. Recombinant protein vaccination, known to induce both humoral and cellular immune responses, has the advantage of lower production costs compared with other platforms and a well-defined production pipeline that can be implemented more easily in different laboratories. However, protein-based vaccines usually result in a stronger humoral response compared with a cellular response and may not be satisfactorily efficient in protecting against intracellular parasites. In contrast, RNA and DNA plasmid vaccines are known to induce a highly efficient cellular immune response but tend to exhibit variable immunogenicity in humans. Plasmid-based strategies still face limitations in terms of uncertainties related to the possibility of genomic integration. Adenovirus-based vaccines are effective platforms due to their ability to induce strong immune responses, particularly CD8^+^ T-cell activation. However, they present important limitations, including reduced efficacy due to pre-existing anti-vector immunity and relatively high reactogenicity [198]. Notably, the COVID-19 vaccine ChAdOx1 developed by AstraZeneca was linked to rare thrombocytopenia, impacting vaccine acceptance and contributing to its discontinuation [199].

As evidenced by vaccine development efforts during the COVID-19 pandemic, the use of combined vaccination platforms may also be advantageous. A large proportion of the global population has received booster doses using various platforms, including recombinant-protein-based, viral-vector-based, and mRNA vaccines. Based on this large-scale experience with COVID-19 vaccines, we could anticipate that prime/boost heterologous regimens that combine mRNA and proteins may be particularly effective as a strategy to control all four NTDs discussed here. Since the mRNA platform offers advantageous features, including rapid adaptability and ease of modification, and may help overcome the typically restricted T-cell responses associated with protein-based vaccines, prime/boost heterologous protocols have been successfully tested in animal models of disease, such as CD. Preclinical studies in mice and dogs have shown promising results using a heterologous vaccination regimen combining plasmid DNA priming with TRASP-adenovirus (Ad5) stimulation, leading to robust protection, primarily associated with CD4^+^ T-cell responses. TRASP comprises different chimeric antigens (B- and T-cell epitopes) derived from two stage-specific TS forms, expressed in the amastigotes and trypomastigotes of *T. cruzi*. However, TRASP-Ad5 efficacy may be limited by pre-existing anti-vector immunity as described above. Nonetheless, recombinant protein vaccination with TRASP antigens, combined with Poly-ICLC, induces both humoral and cellular immune responses more robustly and can protect dogs against *T. cruzi* infection, showing that this is a promising multigenic combination for vaccine development [69]. In addition, an alternative strategy that could be explored for malaria control includes a combination of subunit vaccines (e.g., R21/Matrix-M and RH5.1/Matrix-M) that target different parasite stages [200,201]. Such approaches may help overcome the limitations of current vaccine efficacy and promote broader and longer-lasting immune responses in endemic areas.

## 5. Conclusions

Protozoan NTDs continue to represent a major global health challenge, and the development of effective vaccines has historically been limited by the complex biology and immune evasion strategies of these parasites. Recent advances in genetic manipulation protocols, particularly CRISPR/Cas9-based approaches, have allowed the rational design of GMLAPs, which are now considered promising vaccine candidates. Although preclinical data on GMLAP vaccines indicate that they provide protection levels that are higher compared with previously explored vaccine platforms, crucial challenges remain regarding safety, genetic stability, and large-scale GMP production before approval by regulatory agencies and distribution. However, RNA-based vaccines are more likely to reach clinical testing and, due to their versatility, significant improvements towards better efficacy levels will soon be achieved. Together with improved vaccine technologies, the continued progress in molecular parasitology, including a better understanding of host immune response mechanisms, as well as cost-effective implementation policies in endemic settings, will soon be translated into effective vaccines capable of controlling these and other diseases.

## Figures and Tables

**Table 1:** Number of vaccine candidates in clinical trials for malaria, leishmaniasis, toxoplasmosis, and Chagas disease, according to WHO and ClinicalTrials.gov.

Disease	Status of Vaccine Candidates in Clinical Trials	
	Active	Total
Malaria	38	142
Toxoplasmosis	0	0
Leishmaniasis	1	5
Chagas disease	0	0

Active candidates currently under clinical investigation or candidates in the development pipeline, including completed or not-recruiting trials, and Total: all candidates that have reached clinical evaluation.

**Table 2:** Comparison of the main vaccine platforms with their respective advantages and limitations.

Vaccine Platform	Advantages	Limitations
Protein (subunit)	Highly safe Well-established platform Cost-effective manufacturing Strong humoral responses	Requires adjuvants to boost the immune response Requires multiepitope strategies Limited cellular immunity
DNA (plasmid)	High stability Simple and cost-effective production Variable immunogenicity	Low transfection efficiency in humans Requires nuclear entry Risk of genomic integration
Adenovirus vector	High transduction efficiency, inducing both humoral and cellular responses	Pre-existing immunity (e.g., Ad5) Reactogenicity
mRNA	Highly adaptable platform; possibility to include various antigenic sequences Induces both humoral and cellular responses No risk of genomic integration	Low stability (ultrafreezer maintenance) Dependent on LNP carrier systems
GMLAP	Broad antigen presentation Strong cellular immunity Mimics natural infection Long-lasting immunity	Biosafety concerns Possibility of genetic instability Regulatory challenges Complex manufacturing

GMLAP, genetically modified live-attenuated parasite; LNP, lipid nanoparticle; Ad5, Adenovirus serotype 5.

## Data Availability

No new data were created or analyzed in this study. Data sharing is not applicable to this article.

## Supplementary Materials

The following supporting information can be downloaded at https://www.mdpi.com/article/10.3390/microorganisms14051112/s1, Table S1: Genetic targets in live-attenuated parasite vaccine development, including experimental models, immunization protocols, and protective outcomes. Table S2: Immunogenicity and protective efficacy of mRNA vaccines targeting parasitic diseases.

## Abbreviations

ALM	Autoclaved *L. major*
ATP	adenosine triphosphate
CCD	Chronic Cardiac Disease
CCL or R	C-C motif chemokine ligand or receptor
ChAd63	Chimpanzee Adenovirus serotype 63
CHMI	Controlled Human Malaria Infection
CRISPR/Cas9	Clustered Regularly Interspaced Short Palindromic Repeats–associated/Protein 9
DNA	Deoxyribonucleic Acid
DTUs	Discrete Typing Units
GLP	Good Laboratory Practices
GMP	Good Manufacturing Practices
GMLAP	Genetically Modified Live-Attenuated Parasites
GPI	Glycosylphosphatidylinositol
HIV/AIDS	Human Immunodeficiency Virus/Acquired Immunodeficiency Syndrome
HR	Homologous Recombination
Ig	Immunoglobulin
IL	Interleukin
LNP	Lipid Nanoparticle
MHC	Major Histocompatibility Complex
MVA	Modified Vaccinia Ankara
NANP	sparagine (N)–alanine (A)–asparagine (N)-proline (P) repeat
NK	Natural Killer
NO	Nitric Oxide
NTDs	Neglected Tropical Diseases
PAMPs	Pathogen-Associated Molecular Patterns
PCR	Polymerase Chain Reaction
PEPCK	Phosphoenolpyruvate Carboxykinase
PRRs	Pattern Recognition Receptors
RBCs	Red Blood Cells
RNA	Ribonucleic Acid
ROP	Rhoptry Proteins
ROS	Reactive Oxygen Species
Tfh	T Follicular Helper
Th	T helper
TLRs	Toll-like Receptors
TNF	Tumor Necrosis Factor
TRA	Transmission-Reducing Activity
VLP	Virus-like Particles
WHO	World Health Organization
