# Supplementary material for: Next-Generation Vaccines Against Neglected Diseases: New Promises from Genetically Modified Live-Attenuated Parasites and RNA Vaccines
Source: Microorganisms. 2026 May 14;14(5):1112. doi: 10.3390/microorganisms14051112 (PMC13209279; doi:10.3390/microorganisms14051112)
Supplement: Supplementary file 1 [file microorganisms-14-01112-s001.zip › Supplementary table legends.pdf]

**Supplementary Table S1. Genetic targets in live-attenuated parasite vaccine development, including experimental models, immunization protocols, and protective outcomes.** AMA, amastigotes; BALB/c-nu, BALB/c nude mice; BS, blood stage; BTs, bloodstream trypomastigotes; CHMI, controlled human malaria infection; CSP, circumsporozoite protein; CRF, complement-resistant forms; DTH, delayed-type hypersensitivity; hi, high; IFN-$\gamma$, interferon gamma; IgG, immunoglobulin G; KM, Kunming mouse strain; LS, liver stage; lo, low; MPs, metacyclic promastigotes; MSP1/MSP3, merozoite surface protein 1/3; PCR, polymerase chain reaction; PYS, cyst-derived parasites from Pru strain; RH$\Delta$HXGPRT, RH strain with deletion of the HXGPRT gene; RH$\Delta$ku80, RH strain with ku80 gene deletion; SPZs, sporozoites; TEM, effector memory T cells; TRAP, thrombospondin-related anonymous protein; TRM, tissue-resident memory T cells; TCTs, tissue culture trypomastigotes; Tz, tachyzoites; WT, wild-type. Administration routes: IC, intracardiac; ID, intradermal; IM, intramuscular; IP, intraperitoneal; IV, intravenous; SC, subcutaneous.

**Supplementary Table S2. Immunogenicity and protective efficacy of mRNA vaccines targeting parasitic diseases.**
**Malaria:** PfCSP, *P. falciparum* circumsporozoite protein; NANP, asparagine-alanine-asparagine-proline repeat motif; MIP3$\alpha$, macrophage inflammatory protein 3-alpha; Pfs25, *P. falciparum* surface protein 25; Pvs25, *P. vivax* surface protein 25; PfGARP, *P. falciparum* glutamic acid-rich protein; CelTOS, cell traversal protein for ookinetes and sporozoites; PMIF, Plasmodium macrophage migration inhibitory factor; RPL6, ribosomal protein L6; PyMSP1/8, *P. yoelii* merozoite surface protein 1/8; fCSP, full-length circumsporozoite protein; TRA, transmission-reducing activity; TRM, tissue-resident memory T cells.
**Leishmaniasis:** LinKAP, *L. infantum* kinetoplast-associated protein; PEPCK, phosphoenolpyruvate carboxykinase; DTH, delayed-type hypersensitivity.
**Toxoplasmosis:** GRA6, dense granule protein 6; ROP2A, rhoptry protein 2A; ROP18, rhoptry protein 18; SAG1, surface antigen 1; SAG2A, surface antigen 2A; AMA1, apical membrane antigen 1; VEEV, Venezuelan equine encephalitis virus; MDNP, modified dendrimer nanoparticle; NTPase-II, nucleoside triphosphate hydrolase II; SFV, Semliki Forest virus; MIC13, microneme protein 13; RH, RH strain.
**Chagas disease:** Tc24, flagellar calcium-binding protein; ASP-2, amastigote surface protein 2; TS, trans-sialidase.
**Immunological terms and platforms:** IFN-$\gamma$, interferon gamma; TNF, tumor necrosis factor; IL, interleukin; IgG, immunoglobulin G; NF-$\kappa$B, nuclear factor kappa B; IRF8, interferon regulatory factor 8; Th1, T helper 1; repRNA, replicon RNA; LNP, lipid nanoparticle.
**Administration routes:** ID, intradermal; IM, intramuscular; IV, intravenous; SC, subcutaneous.
